# Review of the Socket Design and Interface Pressure Measurement for Transtibial Prosthesis

**DOI:** 10.1155/2014/849073

**Published:** 2014-08-13

**Authors:** Gh. Pirouzi, N. A. Abu Osman, A. Eshraghi, S. Ali, H. Gholizadeh, W. A. B. Wan Abas

**Affiliations:** Department of Biomedical Engineering, Faculty of Engineering, University of Malaya, 50603 Kuala Lumpur, Malaysia

## Abstract

Socket is an important part of every prosthetic limb as an interface between the residual limb and prosthetic components. Biomechanics of socket-residual limb interface, especially the pressure and force distribution, have effect on patient satisfaction and function. This paper aimed to review and evaluate studies conducted in the last decades on the design of socket, in-socket interface pressure measurement, and socket biomechanics. Literature was searched to find related keywords with transtibial amputation, socket-residual limb interface, socket measurement, socket design, modeling, computational modeling, and suspension system. In accordance with the selection criteria, 19 articles were selected for further analysis. It was revealed that pressure and stress have been studied in the last decaeds, but quantitative evaluations remain inapplicable in clinical settings. This study also illustrates prevailing systems, which may facilitate improvements in socket design for improved quality of life for individuals ambulating with transtibial prosthesis. It is hoped that the review will better facilitate the understanding and determine the clinical relevance of quantitative evaluations.

## 1. Introduction

Amputation constantly remains a cause of concern for individuals, their families, and society. Incidence of lower limb amputation has increased over the years [1–3]. Amputations, particularly of the lower limb, are increasing in developed countries [4]. Traffic accidents, particularly motorcycle accidents [3] and diabetes, are two major causes [5]. Advanced technologies and environmental, social, and economic factors have led to considerable improvements in prostheses. Prosthetic satisfaction is a subjective notion [6]. Prosthetists consider biomechanical parameters, but people with lower limb amputation remain dissatisfied with prostheses [7, 8]. The prime objective for any prosthesis is to provide function in a comfortable manner, but comfort is mainly subjective and difficult to standardize [9, 10]. Comfort primarily involves the pressures between the socket and residual limb. The socket fit, type of prosthetic suspension, and alignment of the prosthesis can alter pressures on the residual limb [11–14].

Essential parts of any prosthesis are socket and suspension mechanism [15]. These parts provide the connection between the residual limb and device. Biomechanical knowledge of the mutual behavior of residual limb, socket, and attachment leads to the improvement of prosthesis functions [16]. Improving the function and comfort will bring satisfaction to patients [17]. After several years of using artificial joint-corset below-knee limb, the patellar tendon bearing (PTB) socket became popular around 1957 [18]. The PTB design demonstrated the concept of concentrated weight bearing. The PTB worked well for approximately 90% of amputees [19]. The concentration of force caused stretch on skin, which is one of the consequences of injury of residual limb. The PTB prosthesis does not completely solve problems encountered in certain conditions such as, short residual limbs.

Two suspension less below-knee prostheses were introduced that seem to offer certain advantages over the PTB prosthesis. Both methods include encapsulation of the residual limb with more intimate connection that eliminate the need for any further accessories, such as belt above the knee. A method was developed by Kuhn in the Research Institute Muenster, Germany, called Kondylen-Bein-Munster (KBM). The method provides high enclosure of the femur and the exposure of the patella. Another method, developed by Guy Taft, introduced in 1968, provided full enclosure of the patella, medial and lateral femoral condyles, called the patellar-tendon-supracondylar (PTS) [20]. Later in 1993, the total surface bearing (TSB) prosthetic socket, ICEROSS, was suggested by Kristinsson. ICEROSS distributes weight on the whole socket surface [21]. Usual prosthetic sockets are a combination of PTB and TSB. However, the popularity of this method paved the way for the innovation of silicon suction socket (3S) [22], which emphasizes the mechanical features of the material. The emergence of high-precision instruments and application of computers led to a new perspective in the socket-residual limb interface. From 1986 to 1987, Steege et al. [23] took research initiatives in this respect. In the latest research, a method was introduced by Sewell at al., which enabled the prosthetist to analyze the pressure distribution of prosthetic socket by inverse problem analysis approach [24].

Numerous questionnaires have been developed as means of evaluating satisfaction with prosthesis and orthosis [25–28]. The Prosthetic Evaluation Questionnaire was introduced to assess satisfaction and problems among users of prosthetic devices. Dillingham et al. [7] conducted a survey and noticed that most of the participants (*n* = 146) were not satisfied with their prostheses due to skin problems and pain. Another study [29] also revealed dissatisfaction with prostheses. Moreover, more than 70% of participants in a study were more satisfied with the pin/lock suspension system than the Pelite liner [30]. On the contrary, in a prospective study, [30] the majority preferred the Pelite liner. Van de Weg and Van der Windt [31] carried out a research on effect of three transtibial systems on satisfaction and found no significant difference. The objective of this study was to summarize the literature addressing prosthetic biomechanics, socket/residual limb interface, socket fit, pressure distribution, shear force, and their effects on user satisfaction.

## 2. Methodology 

### 2.1. Search

EBSCO, Emerald, ProQuest, PubMed, and Web of Science were searched to find related literature from 1980 to 2013. A total of 421 research papers were found. The following keywords, their variations, and combinations were used for the search: prosthesis, lower limb, amputation, transtibial, below knee, socket, pressure, modeling, suspension, and interface.

### 2.2. Criteria

English peer-reviewed papers that addressed socket fit and design, computational modeling, and simulation were selected, which mainly focused on pressure distribution, shear force, and friction.

## 3. Results

A total of 421 research papers were found. The titles of each individual study were assessed. Several articles were excluded as being conducted on dental prosthetics, upper limb prosthetics, and implants, or were common between the databases. After refinement, 75 related papers remained. The abstracts of each individual study were assessed, from which 19 papers were selected for the final review.

### 3.1. Use of Finite Element Methods (FEM) for Socket Pressure Measurement

For years, researchers have used finite element methods to study pressure and stress measurement. The foundations of material and fluid physics, such as hydrostatics and Pascal's law, provide a suitable framework for understanding the residual limb and socket behavior [32]. Another issue is whether mere high pressure causes damage to the tissue [33] or size and intervals (high-frequency events, HFE) are also important. Geometric changes in the muscles and materials are deemed important factors. Findings, such as skin damage caused by “a loading cycle (22–118 times) of 4–23 kPa with a friction coefficient of 0.5” [34], can support this notion because endurance threshold, peak point, and onset of pain are subjective and vary from one person to another. Remarkable ethnic differences also exist in terms of genetics, race, muscle intensity, and skin endurance, thereby lowering the credibility of such experiment results. Such comparisons are ongoing, and the capability of sensors is further enhanced by technological advances, such as the emergence of chips and ultrasensitive fibers [35]. Aside from sensors, computerized modeling has been also considered since 1996.

Similar to an artificial leg, the residual limb is a complicated system with mechanical and biomechanical behaviors. Parameters, such as force distribution, friction, and tension on residual limb against the socket have been investigated through FEM [36]. Tomographic images have also been used to improve 3D FEM modeling. For instance, liner stiffness and its impact on residual limb-socket friction have been evaluated with the solid model constructed using the automesh function of the CAD system [37]. Understanding these variables helps better in the comprehension of mechanical and biomechanical relationships between the socket and residual limb. However, in several cases of modeling, the displacement during donning the socket is neglected [36, 38]. As such, the automatic contact method was applied to overcome these defects [39] to analyze the magnetic resonance imaging (MRI) and skeletal structure using the Mimics software [40].

Software programs, such as SolidWorks (SolidWorks Corporation, MA, USA), Abaqus (Hibbitt, Karlsson & Sorensen, Inc., Pawtucket, RI, USA), magnetic resonance MRI, and XRY Dynamic, have been widely used to understand the biomechanics of suspension systems and residual limb interfaces. After transferring the model and data from Solidworks to Abaqus, FEM assumes that the bones, fat, and muscles are the same elements and form a monolith with different mechanical properties [41]. For the sake of simplicity, the assumption in FEM is that the knee angle does not change with different loadings [40]. Jia et al. studied external and internal parameter and assumed that no motion relationship exists between the residual limb and socket during locomotion [16].

All techniques used for assessing pressure and socket-residual limb tension were aimed at increasing accuracy and producing results approximated to the practical and medical situation. The study on the residual limb-socket interface behavior in a dynamic state has automatically extended the research scope. Some studies have highlighted the mutual effect of the hard and soft tissues of residual limb (e.g., effect of knee movement, changes in the position of residual limb bones, and their function in generating tension and shear forces). The results of these investigations were expected to contribute to practical improvements in socket construction, mainly for the pain management due to socket misfit. Thus, pain perception was evaluated using technical and computerized systems to redesign and rectify the socket prior to actual construction in accordance with the obtained results [42].

### 3.2. Deep-Tissue Damage

Pressure and weight-induced stress, as well as the reaction force of socket-residual limb, may damage the skin. These factors can also cause severe pain due to compression or tension. Several studies have been conducted on deep-tissue damage (DTD) to understand the major causes of such damage [43]. Portnoy et al. conducted a study through 2D and 3D modeling to prepare a preventive model for the DTD in transtibial amputation [44]. Any pressure on the skin affects all internal tissues [43]. The skin has mutual interaction not only with the socket's interface but also with tissues, tibia, fibula, and muscles. The tibia and fibula are parts of the foot and leg set, and all femoral movements and their resultant displacements make it extremely difficult to identify the pressure points in the residual limb [36, 45].

The possibility of evaluating and recording data and their exchange in mechanical and biomechanical evaluation systems, such as FEM, is the main index of this socket design model [46]. By contrast, rectification is a challenging aspect of this method. Extensive research on the use of FEM has covered the features of materials used in the socket and liner, geometric dimensions of the socket-residual limb, and forces and their effects on the configuration of the artificial leg, shear stress intensity, pressure distribution, and friction. The capabilities of existing software products were effectively used to obtain a correct visualization of the mutual residual limb-socket relationship.

### 3.3. Sensing Pressure

Researchers have examined the defects of measuring instruments, weakness of existing sensors in terms of size and sensitivity and impact of heat, which casted doubt on research results. Lee and Zhang aimed to rectify the socket design in a virtual environment before construction through the pain perception threshold as an evaluation criterion [42]. The oral report of the wearer was used as a credible measure for evaluation, rectification, and actual construction of the socket. Additionally, special sensors were utilized to evaluate the pressure between the socket and residual limb and between the liner and socket (Table 2). The most commonly used sensors are fluid-filled transducers, pneumatic transducers, printed circuit sheet sensors, and diaphragm deflection strain-gauge sensors. Modern biofeedback has been used to record the pressure in dynamic and static states. Measurement systems include shear stress neuromuscular system, 3D computer modeling, prototype socket sensor matrices, customized pressure vessels, Rincoe socket fitting system, Tekscan F-Socket pressure measurement system, and novel pliance system. The thickness of these sensors and systems, albeit small, affects the results of studies [47, 48]. The accuracy and response of sensors in curved areas, as well as lumps, have also been compared [48]. The only available system that allows clinical use is smart pyramid called Europa [11, 13, 14]. However, it only provides forces applied below the socket, not the forces or pressure applied inside the prosthetic socket or between the residual limb and liner. Yet, the same technology might be improved to be used inside the socket. The current available pressure mapping systems are complicated and expensive and require laboratory settings. These make it impossible to be used in clinical settings. There is the need to develop portable wireless systems that can be easily used in rehabilitation clinics for objective real-time assessment.

### 3.4. Socket Design and Pressure Distribution

Poonekar (1992) enumerated several factors considered in producing a socket, such as scientific evaluations, possibility of economic production, actual living conditions of person with disabilities, and disability. He likewise highlighted the new technological capacity for developing and using new techniques and materials for lower limb amputations [49]. The adaptation and fitness of existing instruments for prosthetic requirements facilitate the generation of quantitative data on prosthetics. Several techniques, including computer-aided design (CAD) and computer-aided manufacture (CAM), have been also used to reduce the fabrication time of sockets. Undoubtedly, reducing the fabrication time via the CAD/CAM method directly affects the costs [50]. Accordingly, the time is reduced, but it fails to improve the quality of the prosthesis fit [24]. Fabricating duplicate sockets [50] appears irrational because residual limb changes over time and the socket should be rectified only by an experienced prosthetist. Utmost satisfaction is achieved through rectification by prosthetists. The problem of dimensional changes has traditionally been solved by the addition or elimination equal to the change, such as wearing socks. In fact, dissatisfaction with prostheses is mainly caused by strains and injuries associated with socket fit. Sanders et al. found that such changes are difficult to control, predict, minimize, and compare [51]. Their findings showed the effect of time and changes in pressure distribution in the residual limb and the socket interface, as well as increased risk of injuries in residual limb [33].

The human skin does not have the intrinsic capacity to remain undamaged under prosthetic pressure. Naturally, the skin suffers from external or internal forces and undergoes mechanical changes. These changes exert long-term negative effects (albeit minimal) on the skin, which causes reaction and damage, not only to the skin but also to the area underneath [44]. Sanders et al. obtained results that could be used for modeling, such as the association of increased force inside the socket with increased friction between residual limb and socket [59]. They also tested shear forces and concurrently measured the normal state and shear stress with a specialized converter [60] and reported that shear stress damages the residual limb and weakens the skin structure [59].

Computerized and FEM modeling assessments still do not match clinical and prosthetic evaluations for producing a systematic pattern and a reliable index. Such gap exists despite the use of algorithms to rectify final models [61]. Vicon motion analysis (Oxford Metrics, UK) has been also used to assess force in the dynamic phase. The mutual effect of hard and soft tissues of the residual limb has been either taken as fixed or ignored to simplify the equation or computerized assessment. Among these variables are the impact of knee angle and its effect on pressure between the socket and residual limb during walking [62]. Jia et al. aimed to predict these effects via FEM modeling and improve socket rectification [62]. Volume, weight, and thickness of measuring transducers and necessary simultaneous measurement of normal and shear stress necessitated redesigning of transducers [56].

## 4. Discussion 

The systematic investigation of an artificial leg requires quantitative and qualitative data. Various prosthetic components have been investigated in the past. The findings do not completely apply to the clinical settings. Studies have been limited to an increased and improved biomechanical understanding of the interactions among the components of an artificial leg. Quantitative and qualitative studies have not created an accurate and extendable model because of the breadth and plurality of effective parameters in the mutual relationship between the residual limb and the socket [63]. In FEM-based assessments, several modeling parameters are either considered fixed or eliminated based on the preference of the model maker. These assessments approximate reality and identify areas that endure minimum and maximum pressures. Studying a part of the artificial leg and the interaction with the residual limb requires elimination of the effects of other parts. Accordingly, results should be constantly obtained after removing limits and variables. In future studies, the artificial leg should be considered as an integrated system. Moreover, the entire prosthesis should be evaluated in a comprehensive model. It is suggested that new research tools are developed that enable both clinical and laboratory measurements.

All existing studies have been conducted in the context of available sockets. Moreover, a change in performance likely alters existing logic fundamentally. Measuring methods have been applied to the assumption that standard available sockets are used. If new approaches and designs for constructing socket liners are established, then measurement and results would completely change. Application of new technologies requires update and hinges on the expertise of not only prosthetists and physicians but also designers and engineers. In fact, the emergence of new sockets and liners has transformed assessment methods. Prosthetists use these products to solve the problems of patient. Thus, in-depth research improves prosthetists' understanding, thereby increasing patient satisfaction. It can be made possible by developing user-friendly systems for pressure measurement that allows prosthetists evaluating the prosthesis based on simple reports before delivery to the patient.

Several studies have generated favorable results with respect to modeling, as well as experimental, technical, and mechanical equations. However, these results are impractical because of the hypotheses that should be eliminated or taken as fixed in computerized modeling. For instance, modeling friction produces unreliable results because several parameters cannot be accommodated by modeling using the current software. Therefore, the skin and skin subtissues bear the pressure for the moment. In fact, a trade-off exists between the advantages and possibilities of having an artificial leg. Accordingly, all studies should strive to reduce these disadvantages while increasing the advantages to achieve an optimal final product

The increasing popularity of silicon liners has affected the manner by which future evaluations may be conducted. For instance, these evaluations may focus on skin-silicon relationship, advantages and defects, biomechanical performance evaluation, comparison of different liners based on ten years of studies on residual limb-socket interface, pressure, and shear stress. This requires creation of a new discipline, which is different from prosthetics and focuses on production technology, because measuring and modeling software and instruments are constantly being developed. Psychological factors are also among the most important factors that should be taken into account in investigations and experiments on people with lower limb prosthesis [6]. Satisfaction analysis has yielded interesting results in past and opened a new horizon for discussing effective parameters of wearer satisfaction. The effect of the wearer-prosthetist relationship is a major index of user satisfaction [7]. The final aim of the studies is the satisfaction of people with lower limb prosthesis. It can be concluded from the findings that the best model for satisfaction evaluation is the one that integrates both objective data and subjective feedback of the users. One study revealed that different results are obtained despite the same fabrication process and quality used by specialist prosthetists. Hence, satisfaction becomes an internal and psychological matter.

Future studies are expected to deepen understanding of the relationships among vital factors in the design and fabrication of existing sockets. In addition, researchers need to find new and inexpensive solutions that are competitive, both in terms of performance and condition. Since 1945, numerous studies have measured pressure distribution inside the socket for comparative and biomechanical evaluation and for better understanding the residual limb-socket interface [64]. These studies have improved the understanding of the effective parameters in such relationship. However, all employed methods have been shown to possess accuracy constraints. Nevertheless, these studies have shed light on an extremely important aspect, which is the pressure distribution around the residual limb. This pressure distribution directly and indirectly affects the effective indices of user satisfaction, issues relevant to residual limb interaction, and mechanical and biomechanical variables of the socket. The skin has chemical, physical, and mechanical characteristics, which undergo problematic changes when it interacts with the socket and suspension system (Table 1). These changes cause skin damage. Damage and discomfort occasionally occur in blood vessels and circulation [65]. Evaluation and measurement of these changes and heat around the residual limb, as well as correct understanding of this interaction, are important issues [66].

Experts and engineers have sufficiently focused on prosthetics; however, few databases have been created. Compared with databases in other fields, those relevant to prosthetics and orthotics are extremely few, such as National Amputee Statistical Database, RECAL Information Services [67], National Prosthetics Patient Database [68], REHABDATA, Center for International Rehabilitation Research Information and Exchange, and AbleData. Hence, researchers of prosthetics prefer an interdisciplinary approach because of the direct and indirect relationship of this area with other sciences [69]. Finally, rehabilitation is a teamwork consisting of surgeon, physical therapist, occupational therapist, prosthetist, and psychologist [70]. Hence, proper fit may not be achieved if the prosthetist design the most comfortable and intelligent socket, but the surgery and postoperation physiotherapy are insufficient.

## 5. Conclusion

The complexity of the issue, as well as overlapping of engineering and medical parameters, requires cooperation of industrial designers, engineers, and medical experts in a consistent system. Aesthetic parameters should be also considered in this area as part of evaluation index for patient satisfaction. Researchers have studied pressure and stress measurement, but quantitative evaluations remain inapplicable in clinical settings. The central issue is fitting and biomechanical interactions for fit. In addition to the changes in socket fitness over daily activities, attention should be given to the impact of time on residual limb and parametric changes. The time has come to provide better solution to socket design and suspension system through the expertise of specialists, innovative designs, and new measuring instruments. Prosthetic components, such as the pylon, require redesign. In addition, intelligent materials can completely transform the socket design.

## Figures and Tables

**Table 1:** Characteristics of subjects in the studied literature.

Study (*n* = 20)	Age (year)	Cause of amputation (%)	Residual limb length (cm)
Sanders et al. (1992) [34]	23–46	Trauma	13.7 (±2.0)
Sanders et al. (1993) [33]	23–46	Trauma	11–15
Zhang et al. (1996) [36]	Unknown	Unknown	Unknown
Zhang et al. (1998) [45]	43–75	Unknown	Unknown
Sanders et al. (2000) [51]	29–81	Trauma	9–14.5
Goh et al. (2003) [52]	31–62	Trauma-vascular disease	11–15
Lin et al. (2004) [37]	Unknown	Trauma	Unknown
Jia et al. (2004) [16]	56	Unknown	Unknown
Lee et al. (2004) [53]	Unknown	Unknown	Unknown
Sanders et al. (2005) [54]	28–61	Trauma	10.4–20.5
Lee and Zhang (2007) [42]	55	Trauma	Unknown
Portnoy et al. (2008) [44]	29	Trauma	12.76
Wolf et al. (2009) [55]	43–59	Tumor-trauma	Unknown
Abu Osman et al. (2010) [56]	34–77	Trauma-vascular disease	12–17.7
Dumbleton et al. (2009) [57]	25–69	Trauma-vascular disease	10.5–17.5
Boutwell et al. (2012) [58]	43–67	Trauma-vascular disease	Unknown
Kobayashi et al. (2012) [13]	18–60	Trauma-vascular disease	13–15
Kobayashi et al. (2013) [14]	18–61	Trauma-vascular disease	13–15
Boone et al. (2013) [11]	18–61	Trauma-vascular disease	13–16

**Table 2:** Distribution of articles based on the methodology and prosthetic components.

Study	Publication year	Method		Instrument	Socket type	Soft liner type	Measure	Measurement interface
		Static	Dynamic					
Sanders et al. [34]	1992		*✓*	Custom designed transducers, strain gauge	PTB	Pelite liner	Normal and shear stresses	Skin/soft tissue

Sanders et al. [33]	1993		*✓*	Custom designed transducers	PTB	Pelite liner	Normal and shear stresses	Skin/soft tissue

Zhang et al. [36]	1996	*✓*		Simplified model	PTB	Unknown	Frictional action	Skin/soft tissue

Zhang et al. [45]	1998	*✓*	*✓*	Force transducer mounting	PTB TSB	Pelite liner	Stress	Skin/soft tissue

Sanders et al. [51]	2000		*✓*	Force transducer mounting	PTB	Five-ply sock	Pressures and shear stresses	Skin/soft tissue

Goh et al. [52]	2003		*✓*	Force transducer mounting	PCast	Silicone liner	Pressure	Skin/soft tissue

Lin et al. [37]	2004	*✓*		FEM	PTB	Unknown	Pressure	soft tissue/liner

Jia et al. [16]	2004		*✓*	Vicon motion system, FEM	PTB	Unknown	Pressure	Bone-soft tissue

Lee et al. [53]	2004	*✓*		MRI, FEM, Vicon system	PTB	Unknown	Pressure	Soft tissue/socket

Sanders et al. [54]	2005		*✓*	Force transducer	PTB TSB	Neoprene and latex sleeves	Interface stress	Soft tissue/socket

Lee and Zhang [42]	2007	*✓*		Push gauge, FEA	PTB	Pelite liner	Pain threshold pressure	Skin/soft tissue

Portnoy et al. [44]	2008	*✓*		MRI, FEA, pressure sensor	PTB	Unknown	Mechanical conditions, pressure/shear strains	Muscle/soft tissue

Wolf et al. [55]	2009		*✓*	3D gait analysis, pressure sensor, Vicon motion system	TSB	Silicone liner	Pressure	Residual limb/socket interface

Abu Osman et al. [56]	2010	*✓*	*✓*	Transducer, sensor	PTB	No liner	Pressure and shear stress	Residual limb/socket interface

Dumbleton et al. [57]	2009		*✓*	Tekscan F-Scan	PTB	Silicone liner, pelite liner	Pressure	Residual limb/socket interface

Boutwell et al. [58]	2012		*✓*	Motion analysis sensor	PTB TSB	Silicone liner	Gait and pressure	Residual limb/liner interface

Kobayashi et al. [13]	2012		*✓*	Biaxial MEMS tilt sensors, force transducer unit	Unknown	Unknown	Reaction moment	Below the socket

Kobayashi et al. [14]	2013		*✓*	Biaxial MEMS tilt sensors, force transducer unit	Unknown	Unknown	Reaction moment	Below the socket

Boone et al. [11]	2013		*✓*	Biaxial MEMS tilt sensors, force transducer unit	Unknown	Unknown	Reaction moment	Below the socket
